# Neural Specialization for English and Arabic Print in Early Readers

**DOI:** 10.1162/nol_a_00119

**Published:** 2023-12-14

**Authors:** Tracy M. Centanni

**Affiliations:** Department of Psychology, Texas Christian University, Fort Worth, TX, USA; Department of Speech, Language, and Hearing Sciences, University of Florida, Gainesville, FL, USA

**Keywords:** fluency, neuronal recycling, plasticity, specialization, visual word form area

## Abstract

Learning to read requires the specialization of a region in the left fusiform gyrus known as the visual word form area (VWFA). This region, which initially responds to faces and objects, develops specificity for print over a long trajectory of instruction and practice. VWFA neurons may be primed for print because of their pre-literate tuning properties, becoming specialized through top-down feedback mechanisms during learning. However, much of what is known about the VWFA comes from studies of Western orthographies, whose alphabets share common visual characteristics. Far less is known about the development of the VWFA for Arabic, which is a complex orthography and is significantly more difficult to achieve fluency in in reading. In the current study, electroencephalography responses were collected from first grade children in the United Arab Emirates learning to read in both English and Arabic. Children viewed words and false font strings in English and Arabic while performing a vigilance task. The P1 and N1 responses to all stimulus categories were quantified in two occipital and two parietal electrodes as well as the alpha band signal across all four electrodes of interest. Analysis revealed a significantly stronger N1 response to English compared to Arabic and decreased alpha power to Arabic compared to English. These findings suggest a fundamental difference in neural plasticity for these two distinct orthographies, even when instruction is concurrent. Future work is needed to determine whether VWFA specialization for Arabic takes longer than more well-studied orthographies and if differences in reading instruction approaches help accelerate this process.

## INTRODUCTION

Although reading is a human invention, its acquisition to fluency is required in the modern industrialized world. The study of reading acquisition and the brain regions that support this task focus largely on a region on the left fusiform gyrus known as the visual word form area (VWFA; Cohen & Dehaene, 2004; Olulade et al., 2013). The VWFA develops a sensitivity to print as early as age four (Cantlon et al., 2011) such that this region responds to print stimuli more than to baseline conditions. Over the next several years, the VWFA begins to specialize for print and responds more strongly to text than to other forms of visual stimuli (Ben-Shachar et al., 2011; Brem et al., 2010; Cantlon et al., 2011), with this process lasting into early adulthood (Centanni et al., 2017). The level of specialization for print in the VWFA increases as letters become associated with phonological information, likely via connections to the language network (Price & Devlin, 2011; Saygin et al., 2016) and the amount of specialization present is related to word reading ability in children (Ben-Shachar et al., 2011; Centanni et al., 2018; Dehaene et al., 2015) and in formerly illiterate adults (Dehaene et al., 2015). Further, early hypoactivation in this brain region is present in children who go on to a dyslexia diagnosis years later (Centanni et al., 2019). Individuals with dyslexia also lack the specialization gradient that is present in typical readers (Olulade et al., 2015), further emphasizing the importance of this region’s development in fluent reading.

The development of a VWFA requires the brain to allocate neurons to this task. Because reading is a human invention and there is not a dedicated circuit for print present at birth (Saygin et al., 2016), the neuronal recycling hypothesis was proposed as a mechanistic explanation for the specialization of these neurons for print (Dehaene & Cohen, 2007). Prior to the onset of reading instruction, the neurons that will become the VWFA respond to other categories of common visual objects, including faces and tools. With the onset of reading instruction, these neurons begin responding to print and eventually form specialization for print (Cantlon et al., 2011; Centanni et al., 2018; Dehaene-Lambertz et al., 2018; Nordt et al., 2021). It is likely that the baseline firing properties of these neurons make them especially suitable for processing print and are the reason this population of neurons are recycled for the creation of the VWFA. Given the importance of language and linking phonological representations with letter forms, it is important to acknowledge the influence of top-down processes in the development of the VWFA (Price & Devlin, 2011). This influence comes from explicit instruction in schools and in the home and provides the necessary feedback to establish connectivity between speech sounds and letter forms. As such, differences in home literacy environment and instructional practice within and across cultures may impact the development of this neural specialization.

The VWFA is specialized and active for a variety of orthographies (Bai et al., 2011; Baker et al., 2007; Bolger et al., 2005; Heim et al., 2015) and other symbolic stimuli (Martin et al., 2019). Across a variety of alphabetic and logographic languages, the location of the VWFA and its specialization for print are consistent (Bolger et al., 2005) as identified using functional magnetic resonance imaging (fMRI). The VWFA response to print is also detectable in the N170 component of electroencephalography (EEG). The N170 is a leftward negative-going component of the EEG signal that occurs approximately 170 ms after the presentation of print and is a replicated neural marker of the brain’s VWFA as confirmed by combined EEG-fMRI studies (Maurer et al., 2011). The precise temporal precision of EEG is beneficial for studies of reading, allowing for the dissociation of early perceptual responses (such as the P1) from the tuned N170 print response. In 7-year-old children early in the process of learning to read, this print signal (also referred to as the N1) is generally equally strong in response to real words and false fonts (Zhao et al., 2019), and becomes more specialized with age and practice following an inverted-U shaped function (Brem et al., 2009; Maurer et al., 2006; Zhao et al., 2019). Much of the prior work on early specialization for print has been conducted in English. Print systems that are less well studied may differ in key ways that impact the brain’s development of a reading network.

Despite the fact that over 250 million people speak Arabic, children learning to read in this orthography lag behind the global average in fluency tests (Abadzi & Martelli, 2014). This could be, in part, due to the visual complexity of the Arabic script (Al Ghanem & Kearns, 2015; Yassin et al., 2020) and increased cognitive effort required to process this orthography. For example, groups of children who were native Arabic readers were faster at detecting target vowels in a novel orthography (Hebrew) compared to their native script (Abdelhadi et al., 2011). The authors suggest their data support the hypothesis that Arabic text is more visually complex than other writing systems (Carreiras et al., 2013; Yassin et al., 2020), which impacts reading fluency. To date, a few studies have investigated the VWFA’s response to Arabic print, primarily using EEG (Al-Samarraie et al., 2020; Simon et al., 2006; Taha et al., 2013; Taha & Khateb, 2013). These prior studies consistently report an N170 response to Arabic print. Young adults fluent in both Arabic and French exhibited strong and equal N170 responses to both orthographies (Simon et al., 2006), supporting the hypothesis that the VWFA specializes for Arabic in a similar manner as other studied orthographies.

To date, studies of VWFA activation to Arabic print have been conducted in fluent young adults, many of whom were attending university in a Western culture during data collection. It is therefore unknown whether the brain’s development for reading acquisition in Arabic follows the same protracted trajectory as has been shown in English (Centanni et al., 2017). The increased visual complexity of Arabic may require additional cognitive effort early in reading instruction and may impact the development of the VWFA’s preference for Arabic. For example, individual letters in Arabic change shape depending on location within a word and some letters are identical in their base shapes (Saiegh-Haddad & Henkin-Roitfarb, 2014). This is notably different from English, where letter shapes are consistent, but pronunciation depends on context. In addition, Arabic is a language with diacritics; vowel phonemes marked by dots and dashes above or below a letter. While English is an opaque orthography (a single letter may represent several different speech sounds), the transparency of Arabic is different depending on the presence of vowel markings. Given these various levels of complexity, if the visual characteristics of Arabic require more instruction and practice for the VWFA to specialize, it may explain the comparatively lower scores on reading fluency measures and support the use of customized reading instruction strategies to help the brain better tune to Arabic print.

One method for measuring cognitive effort related to reading is through power in the alpha band (8–12 Hz). Reduced power in the alpha band reflects desynchronization of the underlying cortex, which is interpreted as increased cortical activity related to the current task (Pfurtscheller & Lopes Da Silva, 1999). In the context of reading, decreased alpha power is associated with increased difficulty. For example, when reading text passages containing distracting hyperlinks, alpha power was significantly reduced compared to conditions where the text did not contain hyperlinks (Scharinger et al., 2015). In addition, a complementary increase in alpha power is observed when reading is more fluent. Children who were exceptional readers (e.g., a reading level three or more years above their current grade) exhibited increased alpha power when compared to age- and reading-level-matched controls (Suldo et al., 2002). Thus, alpha power may provide another complementary metric for evaluating cognitive effort when learning to read in two unique orthographies.

The goal of the present study was to evaluate the early neural responses to print in young children learning to read in both English and Arabic. This sample of children provides an opportunity to compare neural responses across these two orthographies in a within-subject framework. First grade children in Ras al Khaimah, United Arab Emirates, were recruited and underwent an abbreviated reading assessment as well as a short EEG session. The study was designed to test the hypothesis that, because of the known difficulties in learning to read in Arabic, children would exhibit weaker N170 responses and reduced alpha power to Arabic compared to English even though both orthographies were being taught concurrently. Further, this study tested the hypothesis that a left-lateralized response to Arabic would be present in those with better reading skills.

## MATERIALS AND METHODS

### Participants

Children learning to read in both English and Arabic were recruited from private and public primary schools in Ras al Khaimah, United Arab Emirates. In total, 49 children (*N* = 11 female) were enrolled, of whom 35 were native Arabic speakers. All data were collected in a quiet room in the child’s school or a dedicated space at the Al Qasimi Foundation for Policy Research in May–June of their first-grade year. Thus, all children in the sample had at least one year of formal reading instruction in schools, which included both English and Arabic instruction such that Arabic is the primary language of instruction, with English taught as a second language and used for teaching science. Informed consent was acquired from each parent and verbal assent was given by each child prior to participating. Children who presented for the experiment with hair styles not conducive to the study (braids, ponytails, and pig tails) were asked to take their hair down to participate. Many withdrew, leading partially to the gender imbalance in our sample (*N* = 8 female). The researcher provided instructions in English during data collection. A local research assistant accompanied the researcher and provided translation into Arabic when instructions were unclear to the child as well as administered the Arabic measures. All study procedures were approved by the Texas Christian University Institutional Review Board and children received their choice of a small toy or book for their participation.

### Behavioral Testing

Children first completed two reading tasks in English and two reading tasks in Arabic. In English, children completed single word reading and fluency tasks. In English, children completed the Word Identification subtest of the Woodcock Reading Mastery Test (WRMT-3; Woodcock et al., 2001) and a fluency task. The fluency task consisted of a single 62-word passage from the English Early Grade Reading Assessment (Gove & Wetterberg, 2011) and the number of correct words read within 1 min were noted. If five errors were made, the child was asked to stop reading and was moved on to the next measure. In Arabic, children completed letter identification and fluency tasks. During the letter identification task, children were asked to identify as many Arabic letters and short letter combinations as possible within 1 min. During the reading fluency task, children were given 1 min to read a 49-word paragraph from the Arabic Early Grade Reading Assessment and the child was stopped after five errors were made. Accuracy and reading speed were calculated for each child on each measure. It is important to note that because of time constraints, no IQ testing was completed. Participant demographics and scores for the final sample are reported in Table 1.

**Table 1.** Demographics and scores on all available reading measures reported as mean ± standard deviation for children included in the EEG analyses.

**Measure**	***N* = 44, 8 female**	**Score range**
Age	6.83 ± 0.51	5.4–7.6
WRMT-3 Word ID (raw accuracy, English, *N* = 43)	8.95 ± 8.66	0–29
Words per minute (English, *N* = 42)	18.33 ± 22.17	0–60
Letter Identification (raw accuracy, Arabic, *N* = 33)	38.24 ± 37.13	0–100
Words per minute (Arabic, *N* = 33)	12.42 ± 15.79	0–51

*Note*. Reading accuracy measures (Word ID and Letter ID) are reported as raw number of correct items. Reading speed measures are reported as words per minute. Sample size for each measure is also reported. WRMT-3 = Woodcock Reading Mastery Test.

### EEG Data Collection and Task

All children completed a single EEG task lasting no longer than 20 min. Of the 49 children enrolled in the study, four children withdrew prior to the EEG portion of the study and data could not be collected from one child due to an equipment malfunction. The remaining children were fitted with a 24-channel semi-dry portable EEG system (smarting mobi, mBrainTrain, Belgrade, Serbia) by a trained researcher, and impedances were lowered below 30 kΩ. Data were acquired at a sampling rate of 500 Hz and referenced to the vertex (electrode FCz) with the ground at electrode Fpz using the smarting Streamer 3.4.3 software (mBrainTrain, 2023).

**Figure 1.**
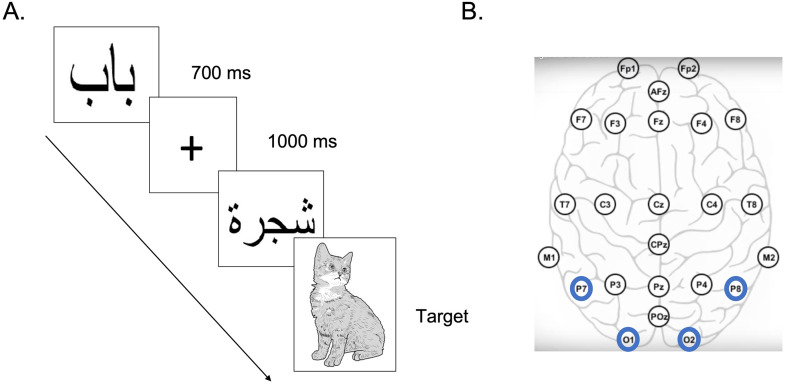
The experimental setup. (A) Children viewed four categories of stimuli (Arabic and English words as well as Arabic and English false font strings) while looking for a picture of a cat. When the cat appeared, children pressed a button on the keyboard. (B) EEG was measured with the mbt Smarting mobi system in the standard 10–20 layout. Electrodes of interest for the current study are highlighted in blue.

A practice block consisted of common objects while the researcher provided instruction and feedback, followed by test blocks containing Arabic words, disconnected Arabic false font strings, English words, and English false font strings (Figure 1A). Children viewed images on a screen and completed a vigilance task in which they pressed a button when a picture of a cat appeared (10% of trials). Children performed well on this task, with an average hit rate of 86.6 ± 9% and reaction time of 572 ± 8 ms. Anecdotally, children often vocalized the word “cat” before or at the same time as pressing the button. All four test stimulus categories contained 10 unique items that were repeated 10 times each. Items were presented for 700 ms with a 1,000 ms fixation cross between each item. Items were presented within blocks in pseudorandom order, with each of the four blocks lasting 3 min. Between blocks, children were given a break to stretch, move around, and prepare for the next block. The entire task lasted approximately 15 min. Stimuli were presented and responses were recorded using custom Python programming (PsychoPy; Peirce et al., 2019).

### EEG Preprocessing and Analysis

All EEG data were preprocessed in MATLAB 2021b (Version 9.11; Mathworks, 2021) using a combination of EEGLab (Brunner et al., 2013) and custom code. Data were filtered with cutoffs of 0.1–30 Hz and then re-referenced offline to an average reference. Eye blink artifacts were identified through independent components analysis and regressed out of the signal. Data were then cleaned to remove movement artifacts and bad channels using the Clean Rawdata plugin (Kothe et al., 2019; transition band [0.25 0.75], bad channels defined as a channel exhibiting a flat line of at least 5 s and/or correlation to their robust estimate based on other channels below 0.8). Interpolation was used to replace bad channels. Data were analyzed from electrodes P7 and O1 in left hemisphere as well as P8 and O2 in right hemisphere (Figure 1B). These electrodes are commonly used to analyze the brain’s print response and were chosen for that purpose (Bach et al., 2013; Lochy et al., 2016). Of note, our electrodes of interest were never in need of replacement due to the heavy emphasis on careful placement and impedance setting of these specific sensors. Epochs were then created for each stimulus category (−500 to 600 ms with respect to stimulus onset) with baseline correction using the 500 ms prior to stimulus onset. Neural data for each participant were included when there were more than 10 good trials available within a given stimulus condition. Of the full sample, 11 children did not contribute data to all four stimulus categories. The final EEG sample thus contained data from 44 children and an average of 37.8 ± 24.9 (*SD*) epochs in response to Arabic false font, 49.6 ± 24.4 epochs to Arabic words, 43.4 ± 27.5 epochs to English false font, and 38.6 ± 25.3 epochs to English words (no main effect of stimulus category; *F*(3, 192) = *p* = 0.09). Given the missing data from various participants in individual stimulus categories, sample sizes for each set of analyses are reported below.

### Statistical Plan

**Figure 2.**
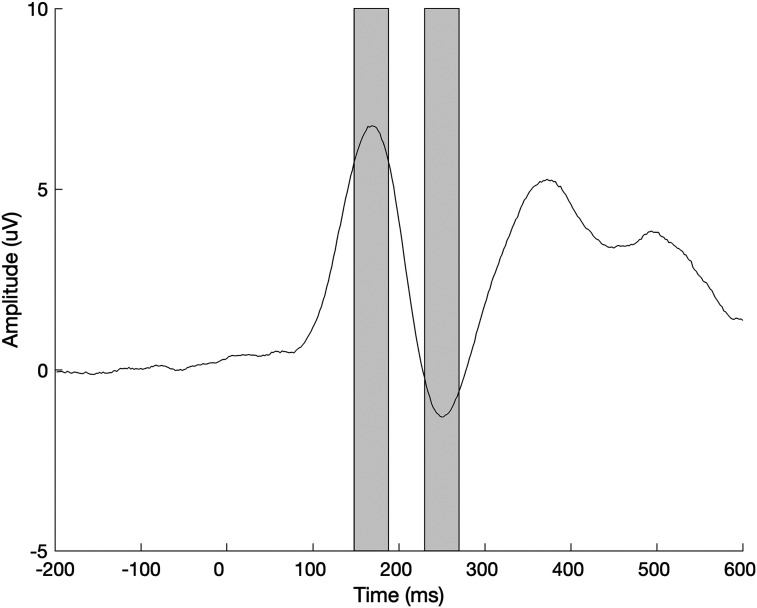
Time windows of interest. Average ERP to all stimulus conditions across all electrodes of interest, used to define time windows of interest for the P1 and N1 components. Gray bars highlight the P1 (148 to 188 ms) and the N1 (228 to 268 ms) time windows of interest.

Repeated measures analysis of variance (ANOVA) were used to evaluate the effects of language, hemisphere, and stimulus type on neural response amplitudes (*N* = 33; the P1 and N1). Time windows for each component were identified by inspection of the average neural response to both word conditions across all electrodes of interest (Figure 2) and defined as a 40 ms window centered on the peak response. The P1 was quantified as the average amplitude 148 to 188 ms post-stimulus onset. The N1 was quantified as the average amplitude 228–268 ms post-stimulus onset. These time windows are in line with prior reports of these peak latencies in early readers (Brem et al., 2013; Eberhard-Moscicka et al., 2016; Maurer et al., 2006). Alpha power was quantified within the 600 ms following stimulus onset in the range of 8–12 Hz in the electrodes of interest. Alpha power was selected as the frequency band of interest because of its prior association with reading tasks (Blohm et al., 2021; Scharinger et al., 2015), including the coordination of eye movement during reading, especially when difficult words are presented (Pan et al., 2023).

Post hoc *t* tests were used to probe any significant effects and interactions. All *t* tests were paired and two-tailed unless otherwise stated. Spearman’s rho was used to evaluate correlations between brain activation and reading scores given the non-Gaussian distribution of reading scores in this sample.

## RESULTS

### P1 Responses

**Figure 3.**
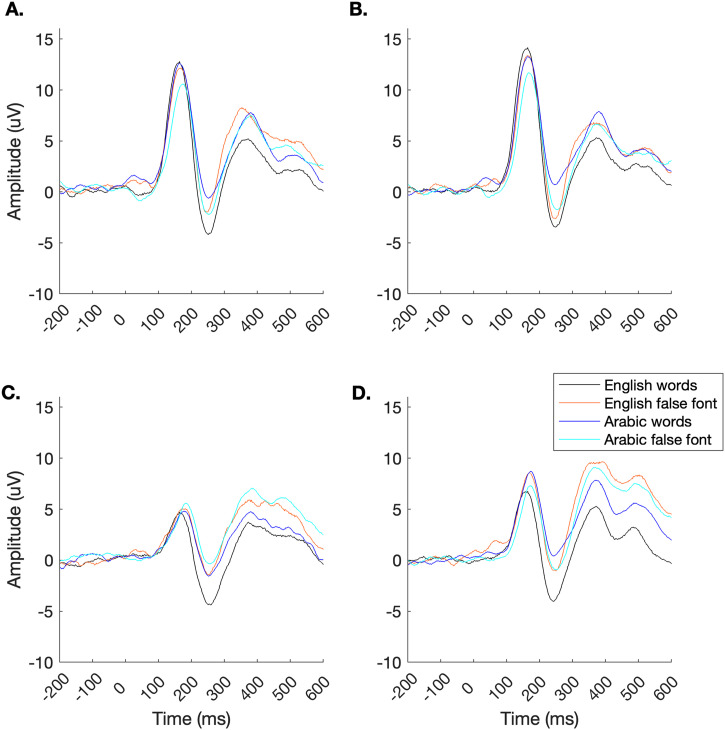
Average ERPs by electrode and stimulus category (*N* = 33). Stronger P1 responses were observed in occipital electrodes (A) O1 and (B) O2, compared to parietal electrodes (C) P7 and (D) P8). English print evoked a stronger N1 response (black lines) compared to Arabic print (blue lines).

**Figure 4.**
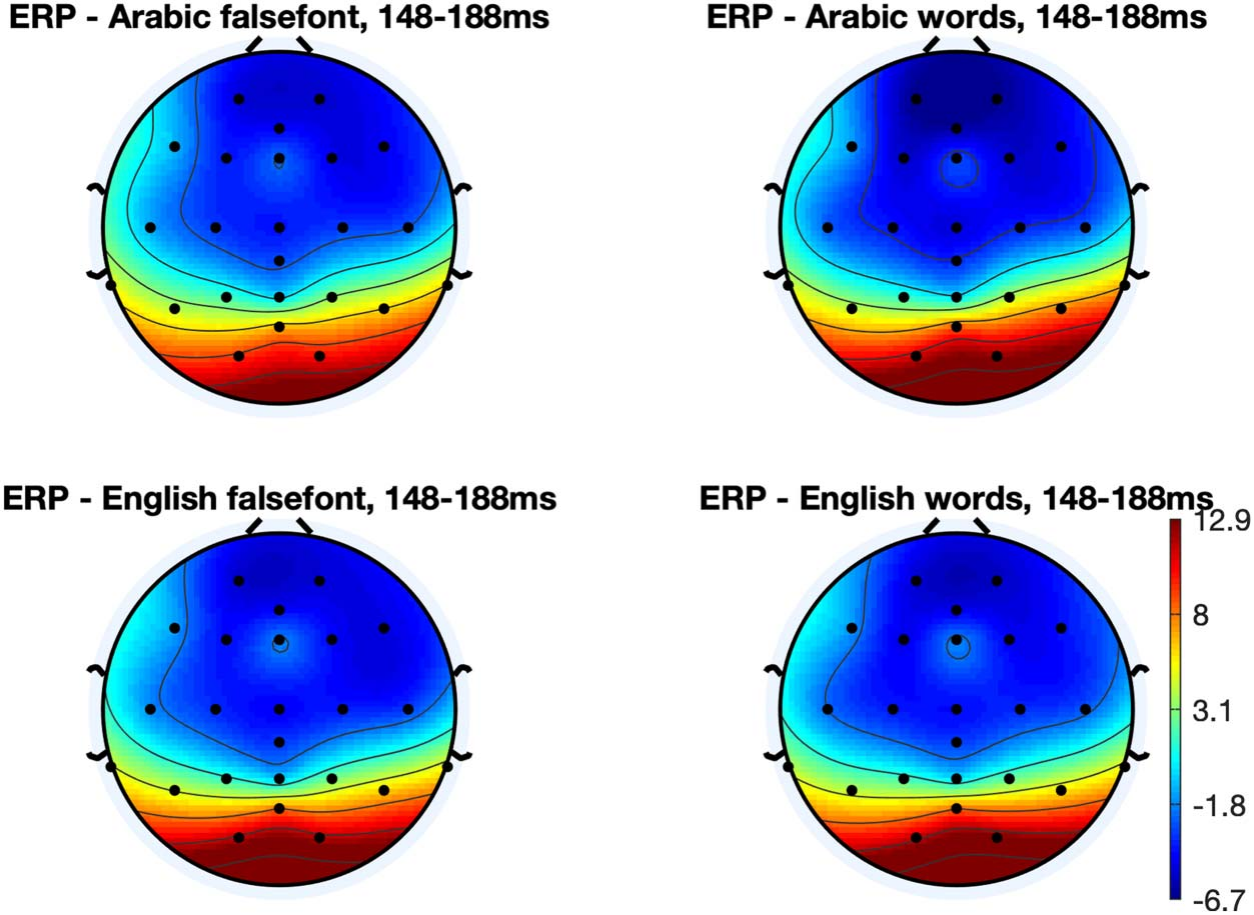
Scalp topographies for the P1 response to words and false font strings in each orthography (*N* = 33).

First, a repeated measures ANOVA was run to investigate effects of orthography (English vs. Arabic), region (parietal vs. occipital) and hemisphere (left vs. right) on P1 amplitude to word stimuli as a marker of the brain’s early sensory response to print (*N* = 33; Figure 3 and Figure 4). There was no main effect of orthography on the P1 (*F*(1, 313) = 0.01, *p* = 0.93). However, there was a significant main effect of region (*F*(1, 313) = 69.68, *p* < 0.0001) such that the occipital electrodes exhibited stronger activation (11.62 ± 1.09) than the parietal electrodes (5.17 ± 0.56; two-tailed, paired *t* test, *t*(42) = 8.17, *p* < 0.0001). There was also a significant main effect of hemisphere (*F*(1, 313) = 6.13, *p* = 0.014) such that the right hemisphere exhibited stronger P1 amplitude (9.50 ± 0.87) than the left hemisphere (7.29 ± 0.91; two-tailed, paired *t* test, *t*(42) = 2.47, *p* = 0.018). There were no interactions (*p*s > 0.31).

### N1 Responses

**Figure 5.**
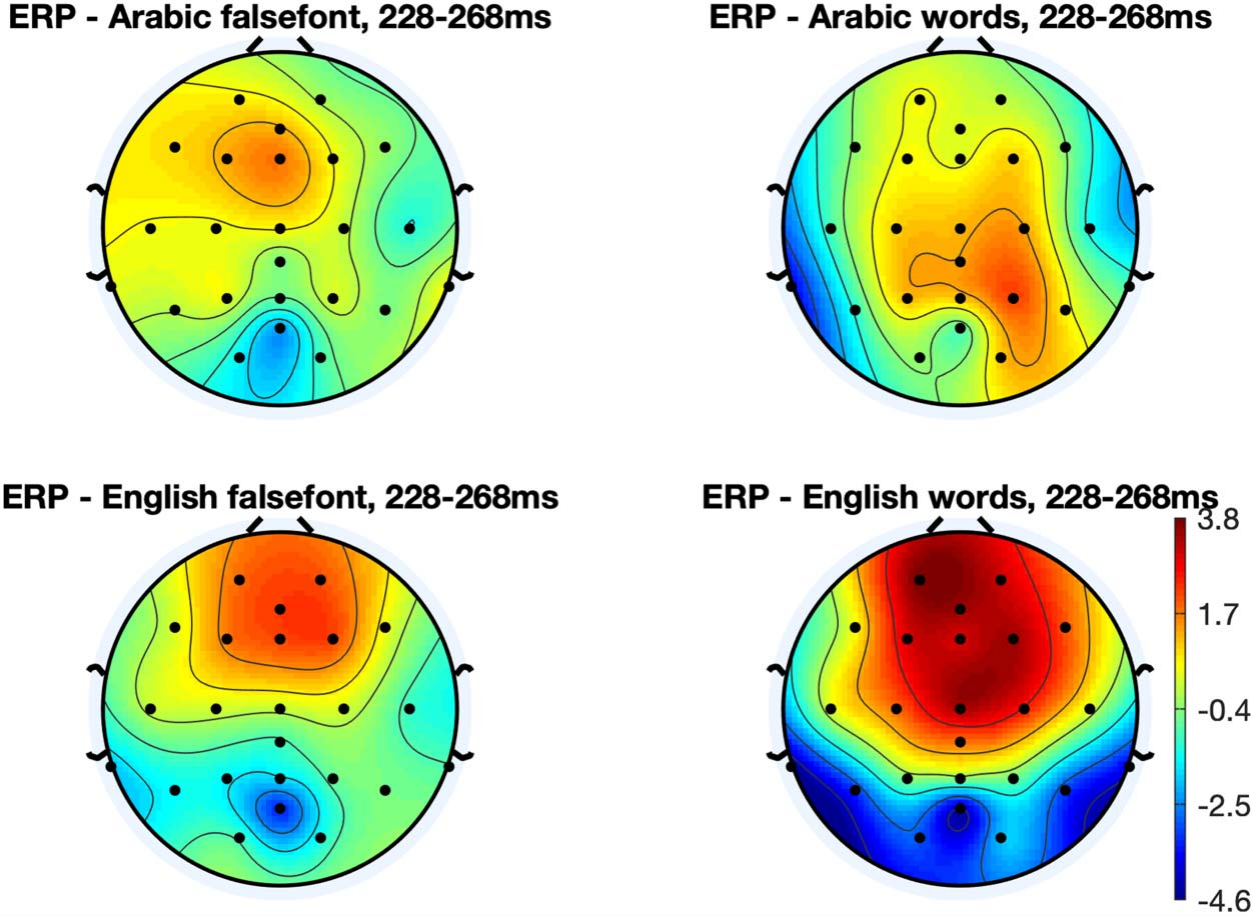
Scalp topographies for the N1 response to words and false font strings in each orthography. N1 amplitude was strongest for English words (*N* = 33).

Another repeated measures ANOVA was then run to investigate effects of orthography (English vs. Arabic), region (parietal vs. occipital) and hemisphere (left vs. right) on N1 amplitude to word stimuli (Figure 3 and Figure 5) as a marker of the brain’s sensitivity for print (*N* = 33). There was a significant main effect of orthography on the N1 (*F*(1, 313) = 33.31, *p* < 0.0001) such that English print elicited a stronger N1 (−3.49 ± 0.73) compared to Arabic print (0.15 ± 0.56; two-tailed, paired *t* test, *t*(36) = 4.82, *p* < 0.0001). There was no main effect of region (*F*(1, 313) = 0.73, *p* = 0.40) and there was a trend in the main effect of hemisphere (*F*(1, 313) = 2.90, *p* = 0.089) such that the left hemisphere exhibited stronger N1 amplitudes (−2.12 ± 0.42) compared to the right hemisphere (−1.04 ± 0.47). There were no interactions (*p*s > 0.43). All electrodes except O2 (*t*(33) = 0.45, *p* = 0.65) exhibited significantly stronger preference for English words compared to English false font strings (paired two-tailed *t* tests vs. 0: *p*s < 0.035). Electrode O2 exhibited a significantly stronger response to Arabic false font than to Arabic words (*t*(33) = 2.84, *p* = 0.008). Responses were marginally stronger for Arabic false font strings compared to Arabic words in O1 (*t*(33) = 2.01, *p* = 0.052) and P8 (*t*(33) = 2.02, *p* = 0.051) but there was no difference at P7 (*t*(33) = 1.73, *p* = 0.092).

### Effect of Orthography

In studies of English reading development, specificity for print develops over time and is associated with reading scores in early readers. To determine whether the same principle is true in Arabic, the amplitude of the N1 was used to quantify specialization for print (N1 response to words − N1 response to false font strings) in each orthography. A repeated measures ANOVA revealed significant main effects of orthography (*N* = 33; *F*(1, 269) = 29.43, *p* < 0.0001) and electrode region (*F*(1, 269) = 9.63, *p* = 0.002) and a trend in the main effect of hemisphere (*F*(1, 269) = 3.57, *p* = 0.059). There were no significant interactions (*p*s > 0.48). When comparing the two orthographies, there was a greater specificity for print in English compared to Arabic in all four electrode locations (Table 2), with three of the four electrode comparisons surviving correction for multiple comparisons (O1, O2, and P8).

**Table 2.** Specificity for print in English vs. Arabic.

**Orthography**	**O1**	**O2**	**P7**	**P8**
English	−2.03 ± 0.82	−0.48 ± 0.96	−3.38 ± 0.92	−3.42 ± 0.95
Arabic	1.74 ± 0.78	2.45 ± 0.78	−1.08 ± 0.56	1.54 ± 0.69
*t* value (*df* = 30)	3.17	2.89	2.22	4.07
*p* value	**0.0035** *	**0.007** *	**0.034**	**0.0003** *

*Note*. Negative values indicate stronger responses to words while positive values indicate stronger responses to false font. **Bold** indicates significant paired, two-tailed *t* test.

* Indicates survival after correction.

**Figure 6.**
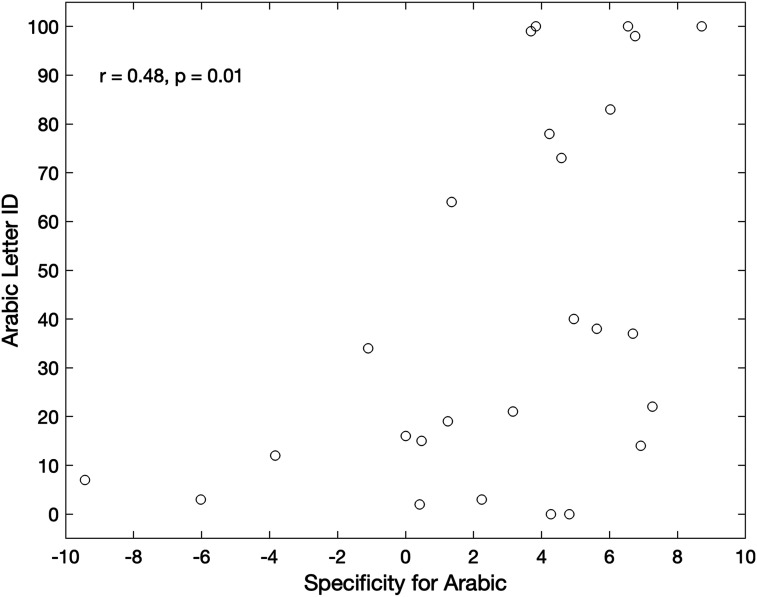
Relationship between neural responses and reading in Arabic. At electrode O2, there was a significant correlation between the specificity for Arabic print (N1 to print − N1 to false font strings) and the letter ID measure such that stronger responses to false font were related to better letter knowledge. Each point represents a single participant. Note that nine children did not have enough data in one of the two stimulus categories to facilitate a specificity calculation and an additional nine children did not complete the letter ID measure. Thus, the correlation was run using data from 26 children.

We next evaluated the relationship between specificity and reading skills. With respect to English, there were no significant correlations at any electrode between specificity and single word reading. There was a trend in the correlation between specificity and reading fluency in O1 (*N* = 34; *r* = −0.31, *p* = 0.08), but no other relationships approached significance (remaining *p*s > 0.13). With respect to Arabic (*N* = 26), neither of the parietal electrodes exhibited any correlation with reading (*p*s > 0.29), nor did O1 (left occipital; *p*s > 0.70). However, specificity for Arabic words (over Arabic false font strings) at O2 (right occipital) was significantly and positively correlated to letter knowledge (*r* = 0.48, *p* = 0.01) and reading fluency (*r* = 0.41, *p* = 0.038). Surprisingly, this correlation was positive, indicating that increased false font responses were associated with better reading. Only the correlation with letter knowledge survived correction (Figure 6). This right hemisphere correlation with reading did not align with the a priori hypothesis of left lateralization.

To further investigate the level of cognitive effort allocated to perception of English words compared to Arabic words, alpha power was quantified in the four electrodes of interest (*N* = 33; O1, O2, P7, and P8). The alpha band has a frequency range of 8–12 Hz and attenuation of alpha power has been associated with cognitive effort (Porta et al., 2010; Roberts & Kraft, 1989) and increased difficulty during print processing (Michel et al., 1994). The average alpha power over this set of electrodes was significantly reduced when viewing Arabic words (3.71 ± 0.54 uV) compared to English words (5.05 ± 0.70 uV; paired, two-tailed *t* test, *t*(43) = 2.33, *p* = 0.024). There were no differences in alpha power across hemispheres for either orthography (*p*s > 0.36). There was a significant difference in alpha power across region, with the parietal electrodes exhibiting less alpha power overall (2.76 ± 0.32 uV) compared to the occipital electrodes (5.99 ± 0.91 uV; *t*(43) = 4.14, *p* < 0.001).

To determine whether alpha suppression and N1 amplitudes reflect similar underlying mechanisms, we utilized Pearson’s correlations to measure the relationship between these metrics. There was no relationship between N1 amplitude and alpha power for responses to Arabic words (*N* = 42; *r* = 0.08, *p* = 0.64). However, there was a relationship between N1 and alpha for responses to English words (*N* = 38; *r* = −0.38, *p* = 0.019) such that more negative N1 responses were associated with increased alpha power. Though these results suggest there may be a relationship between the N1 and alpha power, the restricted use of the four electrodes of interest for this analysis requires these results be interpreted with caution.

## DISCUSSION

The goal of the current study was to investigate the differences in the brain’s print response in early readers simultaneously learning two different orthographies: English and Arabic. First grade children in the United Arab Emirates exhibited stronger N1 responses to English compared to Arabic, and the degree of specialization for print in Arabic was related to reading scores. Further, there was decreased alpha power across the set of parietal and occipital electrodes during exposure to Arabic words compared to English words, suggesting increased cognitive effort. These findings suggest a fundamental difference in the brain’s plasticity for print across two visually distinct orthographies.

### Stronger N1 to English vs. Arabic Print in Early Bilingual Readers

Prior research has established that the VWFA responds to print in a variety of orthographies, including Arabic. Although there is significantly less evidence available to date in Arabic readers, a significant N170 response to Arabic print is present in fluent readers (Simon et al., 2006; Taha et al., 2013; Taha & Khateb, 2013), suggesting that reading in Arabic is supported by the VWFA, as is the case in other orthographies. In fact, the strength of the N170 in a group of bilingual French-Arabic readers was equivalent for both orthographies (Simon et al., 2006), suggesting that in fluent adults, there is no disadvantage for the VWFA to specialize to and respond to Arabic print. The observation of an equally strong N170 to French and Arabic in fluent adult bilinguals suggests that the firing properties of the VWFA, which make these neurons prime targets during neuronal recycling, are also relevant for Arabic. In the current study, there was a stronger N1 response to English compared to Arabic in a group of early readers. This finding may suggest differences in the trajectory for neural plasticity across orthographies. The children in the current sample were largely of Emirati descent and were being educated in schools with a bilingual program. Thus, all children were exposed to English and Arabic daily, with Arabic being the dominant language at home for many of the children. The observation of a stronger N1 to English compared to Arabic in children for whom Arabic was largely their native language suggests not only a fundamental difference in the VWFA’s baseline firing properties but also a longer trajectory for reading acquisition in Arabic compared to other orthographies. Future longitudinal studies are needed to evaluate several possible explanations for the reduced N1 and alpha power to Arabic compared to English in the current sample.

One possible explanation for the reduced N1 in Arabic is that the brain requires additional time and practice to develop specificity for Arabic and may rely on right hemisphere regions to support reading longer than has been shown in English (Maurer et al., 2005). This potentially delayed trajectory suggests there may be something fundamentally unique about Arabic print, as prior work has demonstrated rapid sensitivity of the VWFA to artificial orthographies, including face font and house font (Martin et al., 2019). However, given that the fusiform gyrus contains face and house responsive regions from birth and that these prior studies were conducted in young adults with existing neural reading networks, this comparison to artificial orthographies needs further consideration and may not be applicable to our understanding of Arabic learning. In addition, we did not control for prior exposure to English print in the current study. We therefore cannot rule out the possibility that children received tutoring for English outside school or account for the frequency with which they saw English print in their daily lives.

With respect to baseline firing properties, it could be the case that the visual characteristics of Latin alphabets like English are in better alignment with the tuning of neurons in the eventual VWFA and therefore harness these neurons more easily. Few retrospective studies to date have investigated the baseline firing properties of neurons that go on to become the VWFA. One small longitudinal study of 10 children evaluated fMRI responses at the eventual VWFA location to a variety of visual stimuli (Dehaene-Lambertz et al., 2018). The voxels that eventually became VWFA initially demonstrated a preference for tools, though this preference was somewhat weak, and these voxels were initially not specialized for any single category. The findings from this sample demonstrate that early in schooling, the future VWFA voxels begin responding to both letters and numbers (Dehaene-Lambertz et al., 2018), showing an early preference for symbols that share key visual characteristics, such as straight lines and harsh angles. It is possible that these neurons are not initially well tuned to respond to the letters of Arabic, possibly due to differences in their visual features, namely fewer straight lines and angles and more curves. Future longitudinal research in Arabic-speaking children is needed to understand how neurons in the VWFA specialize for Arabic over time and whether a slightly different population of neurons in the vicinity are ultimately better suited for Arabic. For example, in a sample of English-Chinese bilinguals, distinct regions within the VWFA responded preferentially to a single orthography (Zhan et al., 2023), supporting the hypothesis that different subgroups of neurons within the VWFA are tuned to orthography systems that differ in their visual properties. Such future work would require an imaging technique with better spatial precision, such as fMRI.

With respect to the trajectory of the VWFA, the development of specialization in this region follows an inverted-U shaped pattern related to amount of time spent learning as well as the level of expertise exhibited by the individual, and this developmental pattern could explain the current result. The print specific N1 develops relatively rapidly within the first couple years of reading instruction, with the peak specialization reported around the start of second grade (Fraga-González et al., 2021; Maurer et al., 2006; van de Walle de Ghelcke et al., 2021; Zhao et al., 2019). For example, a cross-sectional study of children learning to read in Chinese revealed similar N1 amplitudes for real words, pseudowords, and false fonts in 7- and 11-year-old children, but stronger responses to words in 9-year olds (Zhao et al., 2019). This inverted-U function has been replicated in longitudinal development studies as well (Fraga-González et al., 2021; Maurer et al., 2006; van de Walle de Ghelcke et al., 2021), suggesting that the peak of N1 specificity for print occurs after the onset of learning, which then decreases with fluency. The current pattern of results could therefore be due to increased practice with Arabic, leading to a decreased N1. This does not, however, explain the reduced alpha power during Arabic print compared to English print, which typically reflects increased cognitive demand, including during reading tasks (Michel et al., 1994; Porta et al., 2010; Roberts & Kraft, 1989; Scharinger et al., 2015). In the current study, most of the children were native Arabic speakers. Although all children were learning to read in both orthographies at the time of data collection, it is likely that there was some variation in the amount of time spent learning and practicing in English versus Arabic, which was unfortunately not noted in the current study. Future work is therefore needed to address the possibility that the difference in EEG responses to Arabic and English is due to training level plasticity differences rather than fundamental differences in the orthographies themselves.

### Impact of Multiple Orthographies on the Reading Brain

The children in the current study were learning to read in both English and Arabic, and so it is critical to consider the impact of multiple orthography learning on the development of the reading brain (Jasińska & Petitto, 2014). English is an opaque or deep orthography, meaning that speech sounds are represented by multiple grapheme(s). In deep orthographies, the reader must process a larger number of graphemes in the same attentional window to accurately map the correct sound to the context of the current word. Thus, deep orthographies have a larger grain size than more shallow orthographies, where the individual grapheme represents a consistent speech sound regardless of context (Lallier & Carreiras, 2018). The difference in grain size may alter the cortical resources needed to read fluently. For example, in a study of English-Spanish bilinguals, there were no differences in activation in reading specific regions, but there were more global connectivity differences that distinguished the two languages (Perfetti et al., 2007).

One study of English-Arabic bilingual children suggested that the two languages do not interfere with each other despite their orthographic differences (Abu-Rabia & Siegel, 2002). However, when considering the initial acquisition of reading, it is important to note that Arabic is a disglossic language, meaning that the spoken version of Arabic (colloquial Arabic) differs from its written form (Modern Standard Arabic). For early readers, this feature of Arabic adds an additional layer of complexity even compared to the difficulty of mastering English orthography. Unfortunately, this is a difficult feature to study, as there is no common written form of spoken colloquial Arabic other than informal text messaging. Thus, it is unclear whether the reduced N1 to Arabic in the current study was due to the added challenge of children learning an entirely new system for reading.

### Right Hemisphere Correlations With Reading

Typical reading development in English is associated with early bilateral processing of print stimuli, followed by a largely leftward lateralization with practice (Yamada et al., 2012). In young pre-readers, specialization for English print (compared with false font) in left VWFA is significantly related to word reading scores (Centanni et al., 2018), suggesting that this leftward lateralization occurs relatively early after reading instruction begins in the United States. In the current study, the leftward N1 to English was present but the leftward N1 to Arabic was essentially nonexistent. Thus, it is possible that the reading network’s tuning to Arabic in these children was at a more premature state compared to the same children’s progress in learning to read in English. If it is the case that the Arabic reading network requires more time for development and is thus premature compared to processing of English, the correlation between right hemisphere false font responses and reading performance is less surprising. In a sample of early English readers, the N170 to words was left lateralized while the N170 to objects was more right lateralized (Sacchi & Laszlo, 2016). Prior work suggests that left-lateralized letter responses and right-lateralized symbol responses are associated with reading and present across languages (Maurer et al., 2005). A rightward lateralization of false font symbols in Arabic could be explained under this assumption.

It is important to note that the current stimulus set included disconnected Arabic false font strings and connected Arabic print. Although both disconnected and connected Arabic leads to N170 activation in fluent adults (Taha et al., 2013), it is possible that in early readers, the brain has not yet linked disconnected letters with the ligatured, connected Arabic commonly used for reading and writing. Additional research is needed in young Arabic readers to elucidate the lateralization of print processing in Arabic and probe the influence of connected text on reading acquisition.

### Limitations

There were four main limitations in the present study that should be acknowledged. First, the initial year of reading instruction for these children occurred when there were still significant pandemic-related disruptions. Many children were attending classes virtually in the fall of their grade 1 year and pandemic protocols were still in place during our data collection window. Thus, it is possible that the development of the reading network was impacted by necessary changes to the standard reading curriculum. Gains in reading dropped significantly in children who experienced virtual learning (Bao et al., 2020; Engzell et al., 2021; Schult et al., 2022). Thus, future work is needed to ensure that pandemic related disruptions did not unfairly impact the typically reading network developmental trajectory for Arabic.

Second, while our Arabic word stimuli were connected, the Arabic false font string stimuli were disconnected. Since Arabic text is typically read in its connected form, it is possible that this difference between the word and false font string stimuli impacted the neural responses to each. A prior event-related potential study demonstrated that the N170 response to connected text was stronger than the response to non-connected text (Taha et al., 2013). Thus, if the connected nature of Arabic words biased the brain toward a larger response, we would have expected to see a strong N1 to words, which was not the case.

Third, The EEG system we utilized here was a low-density, 24-channel system. This system does not contain the spatial resolution to detect and characterize subtle differences in the source of activation. It is therefore possible that the VWFA for Arabic develops in a slightly different location than that for English, and future work using techniques with better spatial precision is needed.

Finally, our sample was skewed with respect to gender, with only eight female participants after removing unusable data points. This skew resulted from a combination of self-selection (we did not target specific children, and recruitment from another country made control of this parameter somewhat difficult) and miscommunication about the need for girls to wear their hair down for the experiment. A language barrier made it difficult to explain this to children and parents in real time. Since this issue was identified prior to consenting, girls with their hair in braids or pigtails who were not willing to take their hair down were not included in the participant count. Future work will learn from this experience and better account for these concerns to recruit a more balanced sample.

## CONCLUSION

The current study was designed to investigate the neural specialization for print in early readers learning both English and Arabic. In this sample of first graders, the N1 response to print was significant for English words but not present in response to Arabic words. Interestingly, specificity to print in English was marginally associated with reading performance and with alpha power in the electrodes of interest. With respect to Arabic, right hemisphere preference for false font strings was associated with letter knowledge. Together, these data suggest there may be fundamental differences in the developmental trajectory for the reading network in Arabic compared to English. Future longitudinal work is needed to better elucidate these differences.

## ACKNOWLEDGMENTS

The authors would like to thank Hanadi Mohammed, Gehad Al Najjar, and Steven Reissig for assistance with logistics and recruitment. We also thank Roa’a Al-Sulaiman for assistance with stimulus creation and translation and Batool Mohsin for assistance with translation and data entry. This work was supported by a generous donation by Dr. Helen Abadzi.

## FUNDING INFORMATION

Tracy M. Centanni, Sheikh Saud bin Saqr Al Qasimi Foundation for Policy Research (https://dx.doi.org/10.13039/100012003).

## DATA AVAILABILITY STATEMENT

De-identified demographics, behavioral data, and neural data are available on OSF (https://doi.org/10.17605/OSF.IO/T7Z8H). Preprocessing and analysis were completed using EEGLab Version 2022.0 (Brunner et al., 2013), and statistics were run and figures were created using MATLAB 2021b (Mathworks, 2021).

## TECHNICAL TERMS

Visual word form area (VWFA): A region on the ventral fusiform gyrus, often in left hemisphere, that exhibits preference in responding to print over other types of visual stimuli.
Electroencephalography (EEG): A noninvasive form of neural imaging that provides millisecond temporal precision and is well tolerated in children.
P1: An early positive peak in the EEG signal, often considered a marker of early sensory processing and familiarity.
N1: An early negative peak in the EEG signal, also referred to as the N170.
Specificity: A measure of print preference in the brain, often calculated as amplitude of N1 to print − amplitude of N1 to control stimulus.
