# Supplementary material for: Neural Specialization for English and Arabic Print in Early Readers
Source: Neurobiol Lang (Camb). 2023 Dec 14;4(4):639–55. doi: 10.1162/nol_a_00119 (PMC10783792; doi:10.1162/nol_a_00119)
Supplement: Supplementary file 1 [file nol-4-4-639-s001.pdf]

Supplemental Information

(Note that the size of stimuli was standardized for size during the experiment)

Arabic word stimuli

أسد (Assad)

كرسي (Kursei)

فيل (Feel)

كتاب (Kitaab)

موز (Moz)

سمكة (Samaka)

شجرة (Shajara)

باب (Baab)

جزر (Jozzar)

قلم (Qalam)

Arabic false font string stimuli

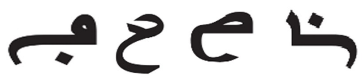

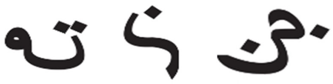

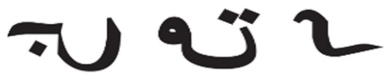

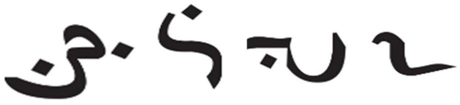

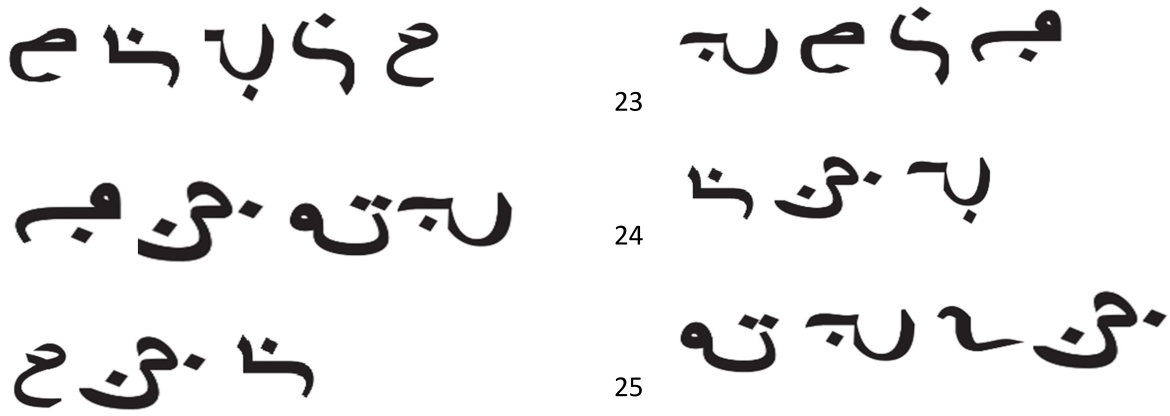

English word stimuli

lion

saw

key

chair

ball

cone

fish

berry

cat

bird

English false font string stimuli

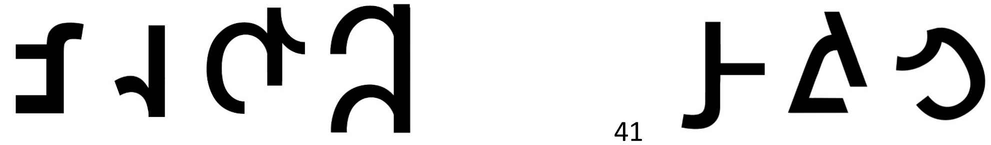

42 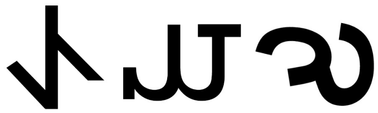

43 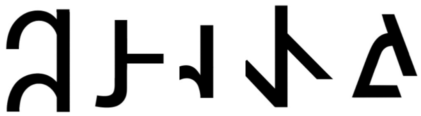

44 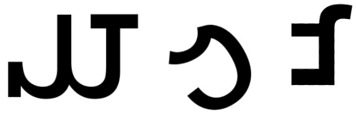

45 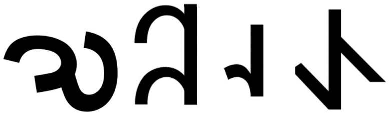

46 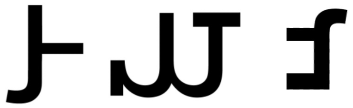

47 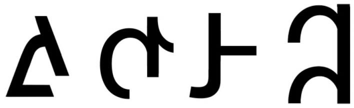

48 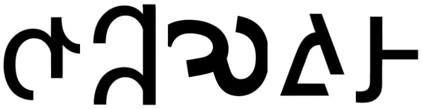

49 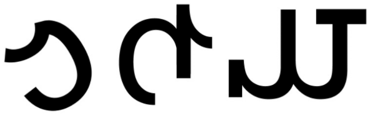

50

51
